# Association of Netrin 1 with hsCRP in Subjects with Obesity and Recent Diagnosis of Type 2 Diabetes

**DOI:** 10.3390/cimb45010010

**Published:** 2022-12-26

**Authors:** Jesús Jonathan Garcia Galindo, Maria G. Ramos-Zavala, Sara Pascoe-Gonzalez, Sandra O. Hernández-González, J. Santiago Delgadillo-Centeno, Fernando Grover-Páez, Alberto Beltrán-Ramírez, Daniel O. Suarez Rico

**Affiliations:** 1 Department of Physiology, Health Science University Center, Universidad de Guadalajara, Guadalajara 44340, Mexico; 2 Institute of Experimental and Clinical Terapeutics, Health Science University Center, Universidad de Guadalajara, Guadalajara 44340, Mexico; 3 Medical Research Unit in Clinical Epidemiology, Specialties Hospital, Medical Unit of High Specialty, West National Medical Center, Mexican Institute of Social Security, Guadalajara 44329, Mexico

**Keywords:** netrin 1, diabetes, obesity, inflammation, high-sensitivity C-reactive protein

## Abstract

Netrin 1 (Ntn1) is a cell migration protein with an anti-inflammatory effect, which may play a key role in the pathological development of type 2 diabetes (T2D). In this study, we evaluate the relationships between the serum concentrations of Ntn1, glucose, and high-sensitivity C-reactive Protein (hsCRP). We carried out a cross-sectional study including 90 individuals divided into three groups (*n* = 30): healthy subjects, individuals with obesity without glucose alterations, and individuals with newly diagnosed T2D. Serum concentrations of Ntn1 and hs-CRP were determined by enzyme-linked immunosorbent assay (ELISA). The serum concentration of Ntn1 was higher in individuals with newly diagnosed T2D (0.33 ± 0.22 ng/mL), in comparison to healthy subjects and individuals with obesity (0.13 ± 0.06 and 0.15 ± 0.07 ng/mL, respectively). In addition, we observed a positive association between the levels of Ntn1 and hsCRP (rho = 0.443; *p* < 0.001) as well as with serum glucose (rho = −0.110; *p* = 0.05). The serum concentration of Ntn1 was higher in individuals with T2D, in comparison with the other groups in this study, and presented a positive correlation with hsCRP. Therefore, Ntn1 can be considered a promising risk biomarker and a potential therapeutic target for T2D.

## 1. Introduction

The combined worldwide prevalence of overweight and obesity from 1980 to 2013 was estimated at 27.5% in the adult population [1]. Overweight and obesity, by themselves, are risk factors for the development of type 2 diabetes (T2D). The prevalence of diabetes in 2019 was calculated to be 9.3% (463 million), which is expected to grow to 10.2% by 2030 (578 million), and to 10.9% (700 million) by 2045 [2].

One of the main risk factors for developing T2D is obesity, which is also a key factor responsible for initiating the inflammatory state [3]. The inflammatory state associated with type 2 diabetes (T2D) may be mediated by acute phase reactants, such as high-sensitivity C-reactive Protein (hsCRP).

The inflammatory process then mediates the recruitment and differentiation of monocytes into the M1 phenotype through Monocytes Chemotactic Protein-1 (MCP-1), thus increasing the inflammatory cytokines and perpetuating the chronic inflammatory state. Ntn1 takes part in the recruitment of monocytes as a guide for cell migration [4].

Ntn1 is a protein that guides cell migration in the neural mapping system, as well as playing a role in the survival and chemotaxis of immune cells. Its activity is strictly related to the binding to Uncoordinated-5-B receptor (Unc5b), which leads to phosphorylation of Peroxisome Proliferator Active to receptors gamma (PPAR-γ), a transcription factor that enhances the expression of adiponectin. In addition, PPAR-γ inhibits the activity of NF-kB and reduces the expression of pro-inflammatory cytokines [5,6,7,8].

Therefore, we hypothesize that the immunomodulatory activities of Ntn1 might play an important role in the pathogenesis and development of insulin resistance, as well as in the onset of and complications related to T2D [9,10,11].

## 2. Materials and Methods

We performed an analytical cross-sectional study. A total of 90 individuals who met inclusion criteria were recruited, at the Instituto de Terapéutica Experimental y Clínica (INTEC) in the Centro Universitario de Ciencias de la Salud of the Universidad de Guadalajara, from 2021 to 2022.

The inclusion criteria were as follows: healthy subjects: 18–35 years old and BMI < 25 kg/m^2^; obesity subjects: 18–40 years old, BMI ≥ 30 but < 34.9 kg/m^2^, without glucose alterations; and newly diagnosed T2D subjects according to American Diabetes Association (ADA) guidelines: 30–59 years old, 25 ≤ BMI < 34.9 kg/m^2^ [3].

Exclusion criteria were as follows: hepatic failure; chronic kidney disease; diagnosis or receiving treatment for coronary, endocrine, rheumatic, and/or neoplastic disorders; acute infectious diseases; use of anti-inflammatory drugs; use of dietary supplements and/or herbal medicine; and pregnancy and breastfeeding; as well as individuals with a diagnosis for COVID-19 or presenting with compatible symptoms for a probable case for COVID-19.

Blood samples were collected from all subjects in red-capped sample tubes in the early hours of the morning with 8 h of prior fasting. The samples were then centrifuged for 10 min at 3000 rpm and the supernatant was collected and stored at −80 °C until further processing.

Weight, height, BMI, and body fat percentage were measured for each patient. BMI was calculated as weight (in kilograms) divided by the square height (in square meters), which was used as a general estimate of obesity. Body fat percentage was evaluated through body bioimpedance measurements collected using a TANITA^TM^ TBF-215 GS.

Ntn1 determination was performed by enzyme-linked immunosorbent assay from MyBioSource (San Diego, CA, USA), which recognizes natural and recombinant human-specific Ntn1, with a variation coefficient lower than 10%. hsCRP was measured similarly, using the ELISA test from MP Biomedicals (USA), diagnostics division Solon, Ohio 44139, catalog number: 07BC-1119.

The normality of data was evaluated using the Kolmogorov–Smirnoff test. The Kruskal–Wallis test was used for non-parametric results involving two or more independent samples; post-hoc analysis was performed using the Tukey test. Correlation analysis was conducted through the Spearman correlation test. Data are presented as means and standard deviations. The significance level to discard the null hypothesis was *p* ≤ 0.05. Statistical analyses were performed using R version 4.1.2 (R Core Team, 2021. R: A language and environment for statistical computing. R Foundation for Statistical Computing, Vienna, Austria. URL https://www.R-project.org/, accessed on 13 January 2022).

## 3. Results

The anthropometric characteristics for all groups are shown in Table 1. Significant differences were observed between groups; for example, the mean age in the newly diagnosed T2D groups was higher than those in the healthy subjects and obesity groups (*p* < 0.001).

We observed differences in HDL (48.5 ± 7.4 vs. 44.5 ± 7.4 vs. 38.9 ± 11.2; *p* = 0.007), triglyceride (112.4 ± 60.2 vs. 115.6 ± 37.6 vs. 194.5 ± 78.9; *p* <0.001), and fasting glucose (80.2 ± 12.6 vs. 77.3 ± 11.4 vs. 118.7 ± 21.4; *p* < 0.001) levels between healthy, obesity, and T2D groups, respectively. The post-hoc test indicated a significant statistical difference between HDL serum concentration in healthy subjects, compared to newly diagnosed T2D (*p* = 0.005), while no statistical difference was found in HDL serum concentration between healthy and obesity groups, or between obesity and newly diagnosed T2D groups (*p* = 0.375 and *p* = 0.151, respectively).

Triglyceride serum concentrations differed between healthy subjects and newly diagnosed T2D groups, as well as between obesity and newly diagnosed T2D groups (*p* < 0.001 in both cases); however, that between healthy subjects and obesity groups did not statistically differ (*p* = 0.985).

No other significant difference, regarding the other cardiometabolic characteristics, was found (Table 2).

**Figure 1:**
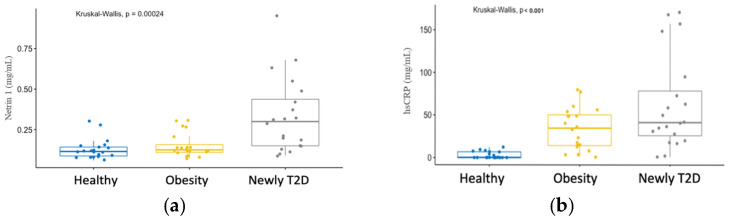
(**a**) Ntn1 serum concentration among study groups; and (**b**) hsCRP serum concentration among study groups. hsCRP, high-sensitivity C-Reactive Protein.

Statistically significant differences (*p* < 0.001) were found in the serum concentration of Ntn1 between the study groups: healthy subjects (0.13 ± 0.06), obesity (0.15 ± 0.07), and newly diagnosed T2D (0.33 ± 0.22). Similarly, significantly different (*p* < 0.001) levels of hsCRP were observed between the groups: healthy subjects (2.9 ± 2.1), obesity (34.1 ± 24.5), and newly diagnosed T2D (62.7 ± 55.5); see Table 3 and Figure 1.

The serum levels of Ntn1 significantly differed between healthy subjects and newly diagnosed T2D groups, as well as between obesity and newly diagnosed T2D groups (*p* < 0.001); however, there was no difference when comparing healthy subjects and obesity groups (*p* = 0.086). Similarly, regarding serum hsCRP, we found a statistically significant difference when we compared the three groups (*p* < 0.001).

The correlations between hsCRP and Ntn1, the clinical variables, and laboratory values are shown in Table 4. Ntn1 serum concentrations showed a positive correlation with hsCRP (rho = 0.443; *p* < 0.001), as well as with glucose serum concentration (rho = −0.110; *p* = 0.05), but not with other variables. On the other hand, hsCRP serum concentrations showed a positive correlation with BMI (rho = 0.670; *p* < 0.001), and visceral fat (rho = 0.555; *p* < 0.001). The rest of the correlations were not statistically significant.

## 4. Discussion

The results of this study indicated that the serum concentration of Ntn1 is higher in subjects with a newly T2D diagnosis, compared with that in obesity and healthy subjects, suggesting an association between glucose serum concentration and inflammation status (evaluated with hsCRP) can develop at the same time.

It has been described that Ntn1 is related to the most common microvascular complications of T2D, such as diabetic retinopathy, in which angiogenesis mediated by HIF-1alpha and Vascular Endothelial Growth Factor (VEGF) is increased, as well as inflammation via NF-kB in the retina, thus explaining the pathophysiology of the disease [11,12]. In diabetic nephropathy, Ntn1 has been proposed as an early biomarker of tubular damage by inflammation with low serum concentrations of this molecule, which may lead to chronic kidney disease accompanied by loss of renal function [13,14].

Other authors, such as Nevada et al., who have measured serum Ntn1 concentration in healthy, obese, pre-diabetic, and diabetic subjects, found even higher Ntn1 serum concentrations in the group composed of healthy subjects, which could be explained by the higher average age in this group, in comparison to ours (49.4 ± 12.1 vs. 22.8 ± 4.3, respectively). However, we agree on the high serum concentration of Ntn1 in individuals with T2D [15].

Yim et al. have also observed a high serum concentration of Ntn1 in individuals with T2D, as well as high Ntn1 in a group composed of individuals with altered fasting blood glucose levels; however, they did not take into account the population with obesity and without insulin resistance [16].

Contrary to our results, Liu et al. have found low levels of Ntn1 in individuals with T2D. They enrolled subjects who were not receiving hypoglycemic treatment at the time of Ntn1 measurement, thus differing from the present study. These differences could be explained by the characteristics of the study population, which was the main limiting factor in our study [17].

Even though there is no currently defined model to explain the increased affinity of Ntn1 and its receptor (Unc5b) in individuals with T2D, we propose a possible explanation for this phenomenon: the existence and development of resistance or loss of affinity may lead to the hypersecretion of Ntn1 without negative feedback, therefore increasing the concentration of pro-inflammatory cytokines and perpetuating the inflammatory status (as measured by serum concentration of hsCRP), which will provoke an increase in NF-kB activity, thus causing more inflammation. All of these mechanisms may produce more damage to the insulin receptor, which will lead to the development of resistance and the onset of T2D and its complications. Therefore, based on the proposed inflammation pattern, we can explain the correlation between increased serum glucose, Ntn1, and hs-CRP concentrations [9,10,18].

Our study has some limitations that should be considered in the interpretation of these results. First, the sample size was relatively modest. Second, being a study with a single measurement, without the follow-up of patients over time nor observation of the evolution of the behavior of Ntn1, we are planning a cohort study in which Ntn1 will be the baseline measurement and including following-up of the variables of interest, giving more weight to the results of this study.

We propose that more research aimed at the pre-diabetic population should be conducted, in order to find the exact point at which Ntn1 levels tend toward higher concentrations, thus increasing its predictive value not only for T2D complications but also for the natural progression of the disease and its prevention through timely interventions.

## 5. Conclusions

Ntn1 levels were found to be significantly higher in individuals with newly diagnosed T2D, as well as being positively correlated with the concentrations of hsCRP and fasting blood glucose.

Ntn1 can thus be proposed as a new risk marker for the development of T2D, which would provide a new tool with which we may evaluate patients with T2D and its relationship to the inflammatory status; which, along with vascular damage, is responsible for the microvascular complications associated to T2D, as well as diabetic retinopathy and diabetic nephropathy. The aforementioned factors are the foremost causes of blindness and chronic kidney disease in our environment, invoking the need for substitutive treatment, and ultimately having direct impacts on our health system and the quality of life of patients.

## Figures and Tables

**Table 1:** Anthropometric characteristics.

	Healthy *n* = 30	Obesity *n* = 30	Newly T2D *n* = 30	*p*
Gender: Male Female	9 (45) 11 (55)	6 (30) 14 (70)	10 (50) 10 (50)	
Age (year)	22.8 ± 4.3 ^1^	21.9 ± 4.5 ^1^	51.3 ± 5.5 ^1^	<0.001
BMI	23.8 ± 2.9	32.7 ± 2.2	30.2 ± 3.9	0.346
Visceral fat (%)	20.3 ± 4.3	33.9 ± 3.2	34.4 ± 10.2	0.249

BMI: Body mass index. ^1^ Statistically significant age difference analyzed by Post-Hoc Tukey test.

**Table 2:** Clinical characteristics.

	Healthy *n* = 30	Obesity *n* = 30	Newly T2D *n* = 30	*p*
SBP (mmHg)	118.00 ± 10.29	121.25 ± 8.79	122.6 ± 11.5	0.346
DBP (mmHg)	75.00 ± 5.72	77.16 ± 6.66	78.6 ± 8.1	0.249
Cholesterol Total (mg/dL)	159 ± 22.62	170.85 ± 22.62	178.2 ± 29.4	0.13
LDL (mg/dL)	93.85 ± 26.18	104.96 ± 21.81	100.3 ± 27.3	0.06
HDL (mg/dL)	48.46 ± 7.43	44.50 ± 7.43	38.9 ± 11.2	0.007
Triglycerides (mg/dL)	112.45 ± 50.20	115.56 ± 37.66	194.5 ± 78.9	<0.001
Glucose (mg/dl)	80.22 ± 12.56	77.25 ± 11.40	118.7 ± 21.4	<0.001

SBP: systolic blood pressure, DBP: diastolic blood pressure, LDL: low-density cholesterol, HDL: high-density cholesterol.

**Table 3:** Serum concentrations of Ntn1 and hsCRP.

	Healthy *n* = 30	Obesity *n* = 30	Newly T2D *n* = 30	*p*
Ntn1 (ng/mL)	0.13 ± 0.06	0.15 ± 0.07	0.33 ± 0.22	<0.001
hsCRP	2.93 ± 2.04	34.13 ± 24.54	62.73 ± 55.46	<0.001

**Table 4:** Correlations between serum concentrations of Ntn1, hsCRP, clinical variables, and laboratory values.

	hsCRP		Netrin 1	
	rho	*p*	rho	*p*
Age	−0.171	0.291	−0.209	0.196
Ntn1	0.443	<0.001	---	---
hsCRP	---	---	0.443	<0.001
SBP	0.093	0.570	0.113	0.489
DBP	0.172	0.289	0.154	0.343
BMI	0.670	<0.001	0.181	0.263
GLU	−0.070	0.667	−0.110	0.050
LDL	0.123	0.450	−0.112	0.490
HDL	−0.055	0.734	0.064	0.693
TG	0.169	0.298	−0.258	0.109
CT	0.175	0.280	−0.182	0.262
Fat	0.555	<0.001	−0.021	0.898

Spearman correlation test. hsCRP: high-sensitivity C-reactive protein, SBP: systolic blood pressure, DBP: diastolic blood pressure, BMI: body mass index, GLU: glucose, LDL: low-density cholesterol, HDL: high-density cholesterol TG: triacylglycerides, CT: cholesterol.

## Data Availability

They are available to any reviewer or reader upon request. Contact the author by correspondence.
